# Comparison of Cytokine Profiles in Ligamentum Flavum from Patients Undergoing Surgery for Lumbar Disc Herniation and Lumbar Spinal Stenosis: An Exploratory Study

**DOI:** 10.3390/cells15141278

**Published:** 2026-07-16

**Authors:** Li-Yu Fay, Chao-Hung Kuo, Yi-Hsuan Kuo, Ching-Jung Chen, Wang-Yu Hsu, Dann-Ying Liou, Ya-Tzu Chen, Kai-Ming Chang, Meng-Jen Lee, Jau-Ching Wu

**Affiliations:** 1 Department of Neurosurgery, Neurological Institute, Taipei Veterans General Hospital, Taipei 112201, Taiwan; leofay1978@gmail.com (L.-Y.F.); chaohungk@gmail.com (C.-H.K.); b101094018@tmu.edu.tw (Y.-H.K.); 2 Institute of Pharmacology, College of Medicine, National Yang Ming Chiao Tung University, Taipei 112304, Taiwan; 3 School of Medicine, College of Medicine, National Yang Ming Chiao Tung University, Taipei 112304, Taiwan; 4 Neural Regeneration Laboratory, Department of Neurosurgery, Neurological Institute, Taipei Veterans General Hospital, Taipei 112201, Taiwan; jirong.chen88@gmail.com (C.-J.C.); wengyu1122@hotmail.com (W.-Y.H.); dragonspruce@gmail.com (D.-Y.L.); yasi0610w@yahoo.com.tw (Y.-T.C.); 5 Department of Molecular Medicine, Koo Foundation Sun Yat-Sen Cancer Center, Taipei 11209, Taiwan; kaiming@kfsyscc.org; 6 Department of Applied Chemistry, Chaoyang University of Technology, Taichung 413310, Taiwan

**Keywords:** intervertebral disc herniation, spinal stenosis, cytokines, ligamentum flavum, hypertrophy, osteoprotegerin

## Abstract

In spinal pathology, lumbar disc herniation (LDH) is typically contrasted with lumbar spinal stenosis (LSS) based on either its pathophysiology (structural compression vs. dynamic/vascular/degenerative changes) or its clinical presentation (acute radiculopathy vs. chronic claudication). We compared the ligamentum flavum (LF) tissue, primary LF cells, and serum cytokine profiles using human cytokine antibody arrays and Western blotting between patients with LDH (*n* = 17) and LSS (*n* = 23). LF specimens underwent hematoxylin–eosin, Masson’s trichrome, and immunohistochemical staining. Compared with LSS, LDH specimens showed relatively preserved LF architecture and higher tissue levels of IL-7, IL-10, eotaxin-3, HGF, and TGF-β2, suggesting an earlier immune-regulatory and reparative phase. In contrast, LSS specimens showed fragmented elastic fibers, increased collagen accumulation, and significantly elevated OPG in primary LF cell cultures. Serum cytokine profiles did not differ significantly between groups. These findings suggest that LDH-associated LF changes may involve immunoregulatory and repair-related cytokine expression, whereas LSS represents a more fibrotic, OPG-enriched remodeling phenotype. TGF-β2, eotaxin-3, and the RANKL/OPG pathway merit further evaluation as stage-specific biomarkers.

## 1. Introduction

Degenerative lumbar spondylosis is one of the most common reasons elderly patients undergo spinal surgery [1]. In the United States, the prevalence of symptomatic lumbar spondylosis is approximately 11%. Despite the availability of conservative treatments, over 600,000 surgeries are performed annually for lumbar spinal stenosis (LSS) [2]. The condition typically arises due to age-related changes such as bone hypertrophy, disc bulging or rupture, and ligamentum flavum (LF) thickening. These changes result in both mechanical compression and secondary ischemic damage to the spinal cord and nerve roots. Clinical manifestations include pain, numbness, paresthesia, weakness, and genitourinary dysfunction [3,4,5].

The LF, located inside the spinal canal, connects the posterior elements of adjacent vertebrae. Under chronic stress, trauma, inflammation, or metabolic disorders, the LF may lose its elasticity and thicken abnormally. This hypertrophy, particularly common in the mobile cervical and lumbar spine, has been observed in approximately 3.8% of the population and can lead to significant neurological deficits [3,4].

Among the proposed mechanisms for LF hypertrophy, inflammation followed by fibrosis appears to play a central role. Several studies have identified biomarkers associated with postoperative pain and disease progression in orthopedic conditions [6]. Although numerous studies have explored biomarkers in ligament ossification, particularly in the posterior longitudinal ligament and LF, no definitive conclusions have been reached [7]. Molecules such as transforming growth factor (TGF)-β, interleukin (IL)-6, and collagens I and III have been implicated in LF hypertrophy pathogenesis, as highlighted by a recent review by Mualem et al. in 2024 [8].

Previous high-throughput transcriptomic or proteomic studies have been conducted on hypertrophied or ossified LF [9,10,11]. However, most of these studies have had small sample sizes (*n* = 3–7), with very little overlap in protein expression across these reports and no consensus on which genes were regulated in diseased LF. Proteins expressed and regulated in cultured spinal ligament cells were not necessarily regulated similarly in vivo. In this study, we used fixed spotted protein arrays to examine the diseased spinal ligament. These protein names were handpicked from the pro-inflammatory, pro-fibrogenic, or immune-regulatory cytokines that were most relevant to the study. This could provide additional information on LF pathology.

## 2. Materials and Methods

### 2.1. Patients and Surgery

**Figure 1:**
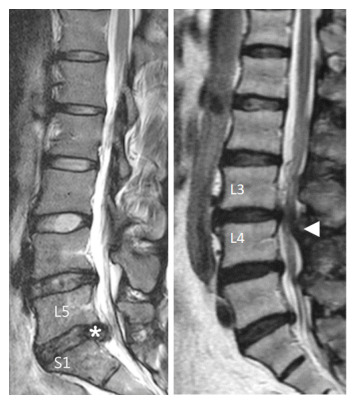
(**Left**): Preoperative MRI, sagittal view, from a 32-year-old LDH female patient who demonstrated spinal stenosis at the L5–S1 level (**left**) and a 55-year-old LSS female patient who demonstrated spinal stenosis at the L3–4 level (**right**). The main cause of stenosis in the LDH patient was from a herniated disc and lesser hypertrophy of the LF. On the other hand, the spinal stenosis in the LSS patient was from severe hypertrophy of the LF. Star: herniated disc. Arrowhead: hypertrophy of LF.

Consecutive patients with lumbar disc herniation (LDH) or lumbar spinal stenosis (LSS) undergoing surgery were recruited between 2021 and 2024. The attending physicians and study nurses provided standardized study information to each candidate. The patient’s participation was voluntary, and declining did not affect clinical care. All patients were provided with informed consent, and the study was approved by the Taipei Veterans General Hospital Institutional Review Board (IRB No. 2021-01-009C). Diagnoses were confirmed using computed tomography (CT), plain radiographs, and magnetic resonance imaging (MRI) (Figure 1). Patients with cerebrovascular disease, coronary artery disease, autoimmune disorders, or other major systemic illnesses were excluded.

In LDH patients, microdiscectomy was performed. Laminotomy was performed to access and resect herniated disc material, and the LF was harvested for analysis. In LSS patients, laminectomy and LF resection were performed, followed by internal fixation in cases of spinal instability. Resected LF specimens (2–15 g) were immediately placed in Hank’s Balanced Salt Solution, HBSS (Life Technologies Corporation, Grand Island, NY, USA) and transported to the laboratory for processing.

### 2.2. Tissue Preparation 

LF tissues were rinsed in HBSS to remove residual blood and stored in a −80 °C refrigerator for later protein extraction and subsequent analysis with Human Cytokine Antibody Arrays and Western blotting (Abcam, Cambridge, UK). Another group of tissues was formalin-fixed and paraffin-embedded (FFPE) for histological and immunohistological analysis.

### 2.3. Blood Sampling

Peripheral blood was collected preoperatively without an anticoagulant. The blood was centrifuged at 2500 revolutions per minute (rpm) for 15 min, and supernatant sera were collected and stored at −80 °C. Before cytokine array analysis, the sera were thawed on ice for 30 min. A total of 500 uL of serum and 500 uL of blocking buffer were mixed to prepare a 2 times-diluted serum sample, and 1 mL was used per array. Cytokine levels were analyzed with Human Cytokine Antibody Arrays (Abcam, Cambridge, UK) (see the following).

### 2.4. Establishment of LF Cell Culture

After thoroughly cleaning the tissue with 0.9% sodium chloride solution, it was cut into small fragments and further washed with serum-free Dulbecco’s Modified Eagle Medium, DMEM (Life Technologies Corporation, Grand Island, NY, USA). The fragments were then immersed in serum-free DMEM containing 0.6% Type 1A collagenase (Sigma-Aldrich, St. Louis, MO, USA). After 80 min of digestion, the supernatant was collected. Serum-containing DMEM was added to terminate the collagenase reaction. The suspension was centrifuged, and the collected LF cells were cultured in DMEM supplemented with 10% fetal calf serum in 10 cm dishes. The culture conditions were 37 °C, 5% CO_2_, and 95% air, with the medium changed every two days. After two weeks, the cells formed a monolayer and were subsequently sub-cultured. Cells from passages 2 to 10 were used for the experiments.

### 2.5. Cell Viability Assay

LF cells were seeded in triplicate in 96-well plates and incubated overnight (24 h) before treatment. Then, 3-(4,5-dimethylthiazol-2-yl)-2,5-diphenyl-2H-tetrazolium bromide (MTT) (Abcam, Cambridge, UK ) dye was added, and the mixture was incubated for 4 h, as previously described. Cell viability was determined by measuring absorbance at wavelengths between 470 and 570 nm.

### 2.6. Protein Extraction

The LF tissue and cell pellet from cultured LF cells were moved to sterile 1.5 mL centrifuge tubes, immediately frozen in liquid nitrogen, and then ground in a mortar. Samples were lysed in NP-40 buffer containing protease inhibitors (250 mM sucrose, 10 mM 4-(2-hydroxyethyl)-1-piperazineethanesulfonic acid (HEPES), 10 mM Tris-HCl, 10 mM KCl, 1% NP-40, 1 mM NaF, 1 mM Na_3_VO_4_, 1 mM ethylenediaminetetraacetic acid (EDTA), 1 mM dithiothreitol (DTT), 0.5 mM Phenylmethylsulfonyl Fluoride (PMSF), and 1 µg/mL of pepstatin, 10 µg/mL of leupeptin, 10 µg/mL of aprotinin) (Merck, Darmstadt, Germany). Lysates were centrifuged at 13,000 rpm for 2 min at 4 °C. Supernatants were collected, protein concentrations were measured, samples were divided into tubes, and samples were stored at −80 °C. Samples were analyzed using Human Cytokine Antibody Arrays (Abcam, Cambridge, UK).

### 2.7. Histological Analysis

Formalin-fixed LF tissues were decalcified using 0.5 M EDTA/Tris-HCl solution for 4–7 days, embedded in paraffin, sectioned at a 4 μm thickness, and stained with hematoxylin and eosin (H&E) (Merck, Darmstadt, Germany) and Masson’s trichrome (Agilent Technologies, Santa Clara, CA, USA). H&E staining was used to evaluate tissue morphology and the degree of calcification (shown by blue-purple deposition). Masson’s trichrome staining highlighted connective tissue, especially collagen fibers, in blue and distinguished it from muscle fibers (red).

### 2.8. Human Cytokine Antibody Array Procedure

Tissues, cell lysates, and serum samples were analyzed using Human Cytokine Antibody Arrays according to the manufacturer’s instructions. Background subtraction was performed using 2 mL of blocking buffer and incubation at room temperature for 30 min. After this, the blocking buffer was removed without rinsing, and 1 mL of serum/buffer mixture or 100 µg of protein samples from tissue or cells were added per array and incubated at 4 °C for 12–16 h. Arrays were washed three times with 1× wash buffer I and three times with 1× wash buffer II (5 min each). A total of 1 mL of biotinylated antibody cocktail was added and incubated for 1.5–2 h at room temperature, followed by additional wash steps. Following this, 2 mL of 1× horseradish peroxidase (HRP)–streptavidin was added and incubated for 2 h at room temperature. After final washes, membranes were placed on blotting paper, treated with 500 µL of chemiluminescent detection buffer, and imaged using a charge-coupled device (CCD)-based imaging system. The optical density was used as an indication of the protein expression level.

### 2.9. Normalization of the Human Cytokine Array Results

A total of 80 cytokine expression levels were obtained for each sample. A total of 6 positive normalization spots and 4 negative spots per array were prepared by the manufacturer. Of the 6 spots, we found that the expression levels of 2 spots fluctuated more than those of the others, with standard deviations >0.15. Therefore, these two spots were eliminated. The remaining 4 spots showed very stable expression levels, with standard deviations of less than 0.09, except for 0.13.

### 2.10. Protein Function Analysis and Clustering on the STRING Website

The expression ratio after normalization was compared between the LDH and LSS groups. The subtracted expression ratio values were used to generate the ranked gene list for the STRING analysis (https://string-db.org/) (accessed on 25 March 2026). The differentially expressed genes (DEGs) were visualized as a network of protein–protein interactions and forced into 3 clusters. For the final figure, the subtracted expression ratio values were adjusted to obtain the optimal color representation of protein up- and down-expression.

### 2.11. Immunohistochemistry (IHC)

Specimens were prepared and sectioned in the same way as those for H&E and Masson’s trichrome staining. Sections were washed with saline and air-dried in a fume hood. They were rinsed in 1× phosphate-buffered saline (PBS) and incubated with blocking serum for 1 h at room temperature. Primary antibodies (anti-osteoprotegerin antibody, Abcam, Cambridge, UK) were diluted in blocking serum and applied overnight at 4 °C. After PBS washes, sections were incubated with secondary antibodies and processed using the Vectastain Elite ABC kit (Vector Labs, Newark, CA, USA). 3,3′-diaminobenzidine (DAB) was used as the chromogen. Stained sections were visualized under an optical microscope (Axioskop 2, Zeiss, Oberkochen, DE, Germany).

### 2.12. Western Blotting

Total protein was extracted from the LDH and LSS specimens, as described for the cytokine array above. A total of 5 µg of protein from the cell lysate was analyzed by Western blot using 10% or 12% sodium dodecyl sulfate–polyacrylamide gel electrophoresis (SDS-PAGE). The gels were then transferred to PVDF membranes (Millipore Corp., Burlington, MA, USA) and incubated overnight at 4 °C with antibodies against the osteopontin and osteoprotegerin (Abcam, Cambridge, UK), IL-7, IL-10, HGF, Flt3-ligand, Eotaxin3, and TGF-β2 antibodies (GeneTex, Irvine, CA, USA), followed by a horseradish peroxidase-conjugated secondary antibody for 1 h at room temperature. Immunoreactivity was visualized using enhanced chemiluminescent detection (Perkin Elmer Co., Waltham, MA, USA).

### 2.13. Measurement of Ligamentum Flavum (LF) Thickness

An axial T1-weighted magnetic resonance image was obtained at the facet joint level of the lesion. The thickness of the ligamentum flavum was traced using the manual cursor technique as described by Park et al. MRI images were acquired, and image analysis, including region-of-interest measurements, was performed using a PACS workstation (SmartIris, Taiwan Electronic Data Processing Co., Taipei, Taiwan).

### 2.14. Statistical Analysis

For the human protein array data, multiple-comparison adjustment was performed using the false discovery rate (FDR) method. Differential protein expression was evaluated using Welch’s *t*-test, the Mann–Whitney U test, and the limma moderated *t*-test; however, none of these analyses identified significantly differentially expressed proteins after FDR correction (Supplementary Files S1–S3). Therefore, as an exploratory approach, proteins were ranked according to a standardized effect score. The highest-ranking candidate proteins were subsequently selected for validation by Western blotting.

### 2.15. Use of Artificial Intelligence Tools

Artificial intelligence (AI)-assisted tools were used solely to support literature retrieval, preliminary drafting of selected sections of the text, and English-language editing. No AI tools were used for data analysis, data interpretation, or the generation of scientific conclusions. All outputs generated with AI assistance were critically evaluated, edited, and approved by the authors. The authors retain full responsibility for the accuracy, originality, and integrity of the work.

## 3. Results

### 3.1. Patient Demographics and LF Morphology

This study recruited 40 patients who received surgical treatments. A total of 17 of them were treated for LDH, and the other 23 were treated for LSS. The mean ages of the patients with LDH and LSS were 48.9 ± 11.9 and 59.1 ± 9.5 years, respectively. The age of the LSS group was significantly older than that of the LDH group (*p* < 0.05) (Table 1). Surgically obtained LF from LDH patients was used as a clinically available comparator representing less advanced LF remodeling than LSS. This strategy has precedent in LF studies [12,13,14]. As a result, age may confound part of the observed differences. The number of samples used for each preparation is listed in Table 2. The male-to-female ratio in both groups was similar and not significantly different (*p* = 0.91). On MRI, LF hypertrophy was less prominent in LDH; in LSS, hypertrophied LF encroached on the canal, compressing the thecal sac (Figure 1).

### 3.2. Cytokine Expression in LF Tissue

**Figure 2:**
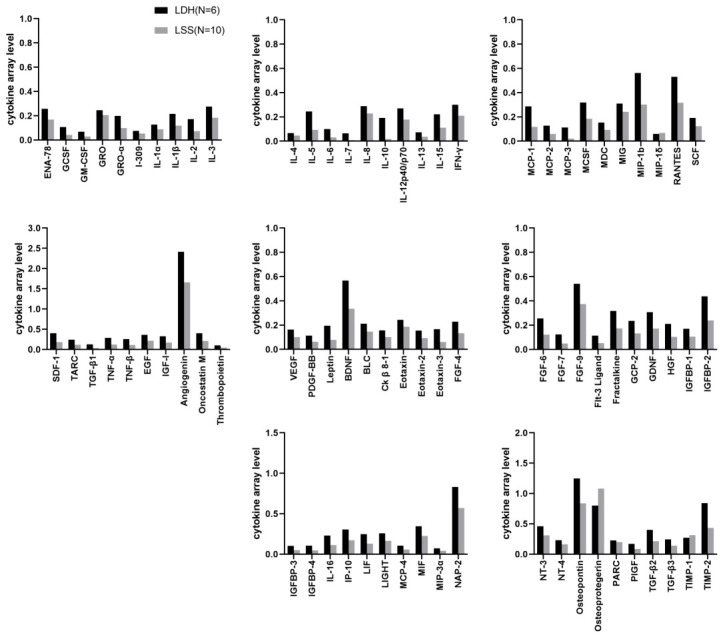
Cytokine expression profiles in the LF tissue in patients with LDH and LSS. IL-7 and IL-10 were found to be higher in the LF tissue of LDH patients than in those of LSS patients. In addition, eotaxin-3, Flt-3-ligand (Flt3L), hepatocyte growth factor (HGF), and TGF-β2 were also higher in the LF tissue of LDH patients than that of LSS patients. Other cytokines showed no differences in LF tissue between the two patient groups.

The cytokine expression in LF tissue from patients with LDH and LSS was compared. The levels of pro-inflammatory cytokines, such as tumor necrosis factor (TNF)-α, IL-1β, and IL-6, were not significantly different between the two groups (Figure 2). The levels of IL-7, IL-10, eotaxin-3, Flt-3-ligand (Flt3L), hepatocyte growth factor (HGF), and TGF-β2 in LF tissue were significantly higher in LDH patients than in LSS patients, or conversely, they were lower in LSS patients. These results are also summarized in Table 3.

**Figure 3:**
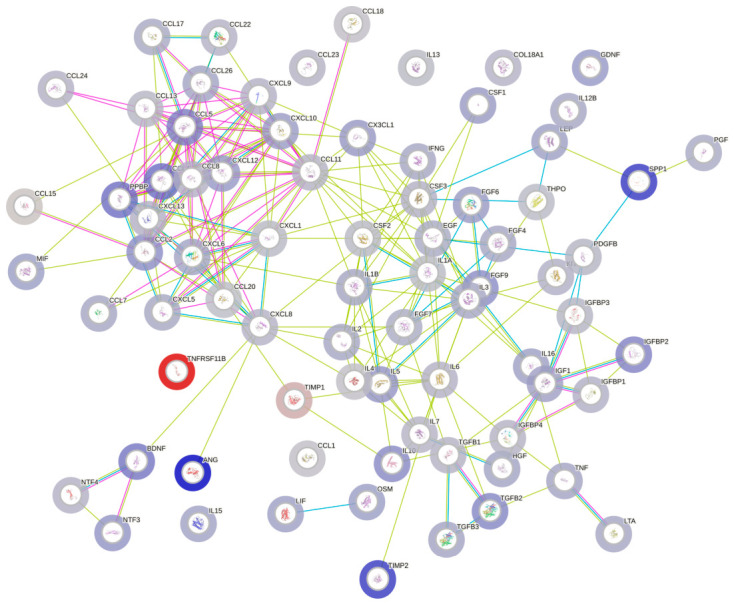
The expression ratios were compared between the LDH and LSS groups. The subtracted expression ratio values were used to generate the ranked gene list for the STRING analysis (https://string-db.org/) (accessed on 25 March 2026). The differentially expressed genes (DEGs) were visualized as a network of protein–protein interactions. The top expressor of LDH/LSS was marked in blue around the bubble, and the top expressor of LSS/LDH was marked in red. ANG: angiogenin, SPP1: osteopontin, TNFRSF11B: osteoprotegerin.

The gene expression and functions were analyzed using g:Profiler (https://biit.cs.ut.ee/gprofiler/) and the STRING website (accessed on 25 March 2026). In the g:Profiler analysis, because the cytokine array we used was purposely designed to contain mostly cytokines relevant to immune and growth responses, most of the proteins were densely clustered in the same molecular function (MF) and biological process (BP) categories. We later used a ranked gene expression list as the dataset in the STRING website (see Supplementary File S4). Protein interactions were mapped with different colors, and genes/proteins were clustered. The top-expressed protein levels do not align with our cytokine array results, as their expression varied and did not differ statistically between the two groups. However, they remained relevant, and their expression and functional interactions are shown in the STRING protein-interaction map (Figure 3). The top three differentially expressed proteins (LDH > LSS) were angiogenin (gene name = ANG), osteopontin (gene name = SPP1), and TIMP2, as demonstrated by the deepest blue circle around the bubbles. The top three differentially expressed proteins (LSS > LDH) were osteoprotegerin (gene name = TNFRSF11B), TIMP1, and CCL15. Without any assumptions, these 80 cytokines clustered into 64 groups, indicating they were not particularly clustered. This is reasonable as the platform was rich in cytokines with a specific functional repertoire. When the functions were forced to cluster into three groups, these 80 proteins formed three groups: the largest group consisted of chemoattractant cytokines (upper-left of Figure 3), then immunoregulatory cytokines (right half of Figure 3), and finally neurotrophic factors (mostly bottom-left of Figure 3).

### 3.3. Cytokine Expression in the Cultured LF Cells

**Figure 4:**
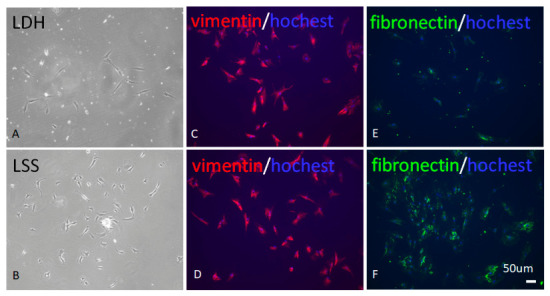
The immunofluorescence stain of primary cultured LF cells from LDH (top row) and LSS (bottom row). Vimentin was stained with appropriate first and secondary antibody conjugated with cy3, fibronectin stained with Alexa Fluor 488, and the nucleus stained withHoechest (blue in color). (**A**) LDH cells photographed in phase contrast; (**B**) LDH cells photographed in phase contrast. (**C**) The same LDH cells stained with vimentin and hochest. (**D**) The same LSS cells stained with vimentin and hochest. (**E**) The same LDH cells stained with fibronectin and hochest. (**F**) The same LSS cells stained with fibronectin and hochest. The elongated, spindle-shaped, or fusiform-shaped cells can be seen in phase pictures (left column). They were positive for vimentin and fibronectin on immunofluorescence staining (magnification: 200×, scale bar: 50 µm).

The human cytokine array was used to analyze the primary cells cultured from LF tissues obtained from the LDH and LSS patients (Figure 4). They were scattered, elongated, spindle-shaped, or fusiform-shaped cells, with visible elongated processes that were most likely fibroblast-like or mesenchymal cells (Figure 4). They were positive with vimentin and fibronectin under immunofluorescence staining (Figure 4).

**Figure 5:**
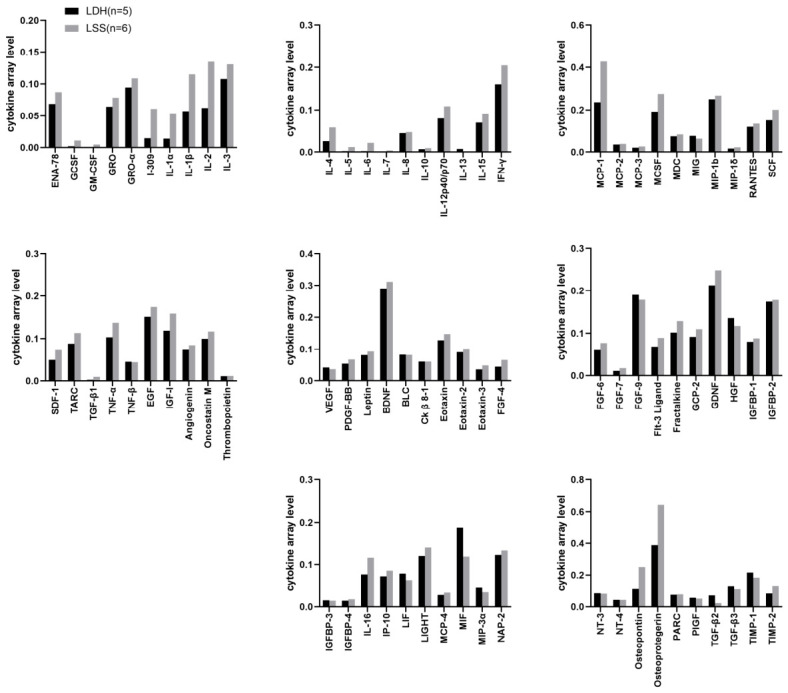
Cytokine expression in primary cultured LF cells from patients with LDH and LSS. OPG expression was higher in primary cultured cells of LSS patients than in those of LDH patients in the cytokine array.

**Figure 6:**
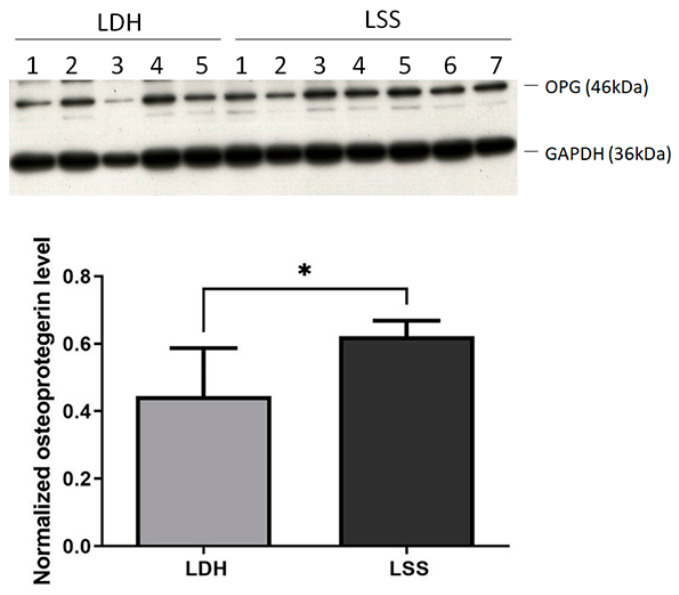
Cytokine expression in primary cultured LF cells from patients with LDH and LSS. OPG expression was further confirmed by a Western blot. * indicates statistical significance at *p* < 0.05.

These primary cells from LSS patients exhibited significantly higher levels of osteoprotegerin (OPG) than those from the LDH patients (Figure 5 and Figure 6). The mean OPG expression in primary LF cell cultures showed the largest intergroup difference, with higher levels in LSS than in LDH. The top differentially expressed gene (LSS > LDH) in the uncultured tissue list was also osteoprotegerin (gene name: TNFRSF11B; see Figure 3). We further assessed OPG expression in cultured primary cells from both groups using Western blot, and the level was higher in the LSS group than in the LDH group (Figure 6). This also corroborated the results from the tissue protein array.

### 3.4. Serum Cytokine Expression

The serum samples showed no significant differences between the LDH and LSS groups across 80 cytokines tested with the human cytokine array. Many pro-inflammatory cytokines that were reported to be significantly elevated in patients with degenerative disc disease or spinal stenosis were not differentially expressed. The leptin level, which has been reported to be associated with spinal ligament ossification, differed but not significantly (*p* = 0.41). Compared with a normal control (i.e., a healthy volunteer), many proteins were significantly elevated in both groups (see Supplementary Files S5 and S6, for details).

### 3.5. OPG Expression on LF Tissue Sections

**Figure 7:**
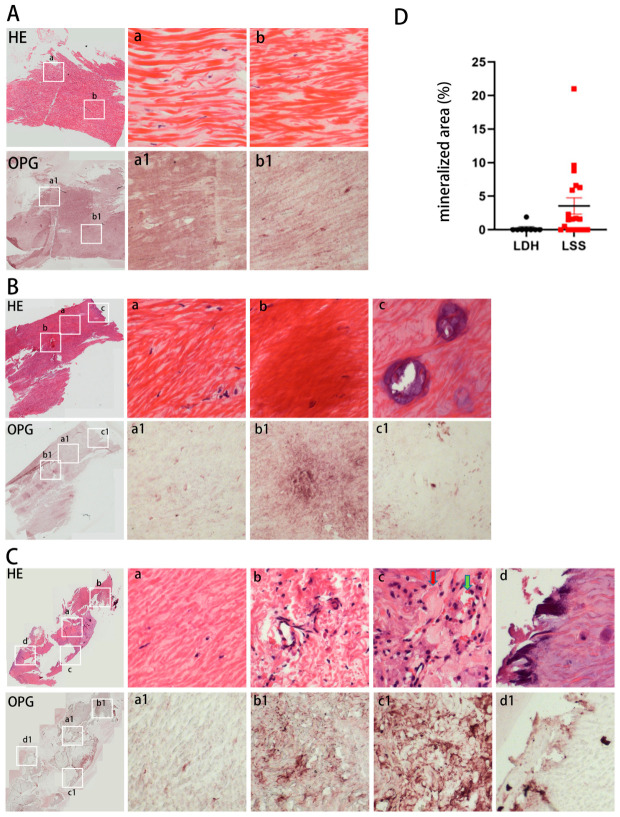
Sections were stained with H&E staining (upper row, labeled HE, magnification 20×), and adjacent sections were stained with OPG IHC (lower row, labeled OPG, magnification 20×). White squares in HE sections were enlarged to show details (labeled a,b,c,d, magnification 200×). White squares in OPG sections were enlarged to show details (labeled a1,b1,c1,d1, magnification 200×). (**A**) The LF tissue from a 36-year-old LDH female. (**B**) The LF tissue from a 57-year-old LSS male. (**C**) The LF tissue from a 59-year-old LSS male. Note that an extra stage with very light pink circles that were devoid of a nucleus (red arrow, C-c), and the presence of many nuclei around the cavity that have the morphology of blood vessels around the cavity (green arrow, C-c). (**D**) Calcified regions on H&E-stained sections were quantified using ImageJ. The percentage of calcified area was negligible in LF from LDH, whereas LSS specimens showed wide variability (mean = 0.23 ± 0.67% vs. 4.06 ± 6.61%, respectively, *p* = 0.048).

The elastic fibers were rich in proteoglycans and stained faintly eosinophilic on H&E. The relatively healthy LF was homologous and rich in elastic fibers that were arranged in order. The OPG IHC staining demonstrated that OPG was not present in the areas of healthy elastic tissue. In spinal ligament pathology, a stage of calcification that follows fibroblastic ligament proliferation and precedes overt ossification can be demonstrated by blue-purple precipitation around differentiating chondroblasts/osteoblasts [15]. In LDH patients with relatively healthy ligaments, the whole tissue was homologous and rich in elastic fibers that were arranged in order (Figure 7(A-a)), some with elevated red staining (Figure 7(A-b)). OPG is expressed during the developmental stage of bone formation as well as in bone regeneration and, in this case, served as a marker of the potential for bone formation. OPG was not present in the areas of healthy elastic tissue (Figure 7(A-a1,A-b1)).

As for the LSS tissues, two morphological types were observed. Both had three stages: (1) Proteoglycan deposition of the elastic fibers, demonstrated by red colors in HE staining (Figure 7(B-a,C-a)), without OPG expression in the same area (Figure 7(B-a1,C-a1)). (2) Dysregulated red-colored fibers (Figure 7(B-b,C-b)), and the presence of OPG in the same area (Figure 7(B-b1,C-b1)). (3) Deep blue-purple extracellular deposition (Figure 7(B-c,C-d)), indicating calcification around several cells with larger sized nuclei, and absence of OPG expression (Figure 7(B-c1,C-d1)). 7C-c shows an extra stage with very light pink circles that were devoid of a nucleus (red arrow, Figure 7(C-c)), and the presence of many nuclei that have the morphology of blood vessels around the cavity (green arrow, Figure 7(C-c)). The pink circles were devoid of OPG staining, whereas the area with numerous cells surrounding the blood vessels was highly OPG-positive (Figure 7(C-c1)). The area of calcification, as indicated by the blue-purple color on HE, was analyzed using the ImageJ software (1.48v). The percentage of calcified area in LF tissue from LDH patients was negligible, while that from LSS patients was significantly higher and varied widely among patients (mean = 0.23 ± 0.67% vs. 4.06 ± 6.61%, respectively; *p* = 0.048) (Figure 7D).

### 3.6. Western Blot Analysis

**Figure 8:**
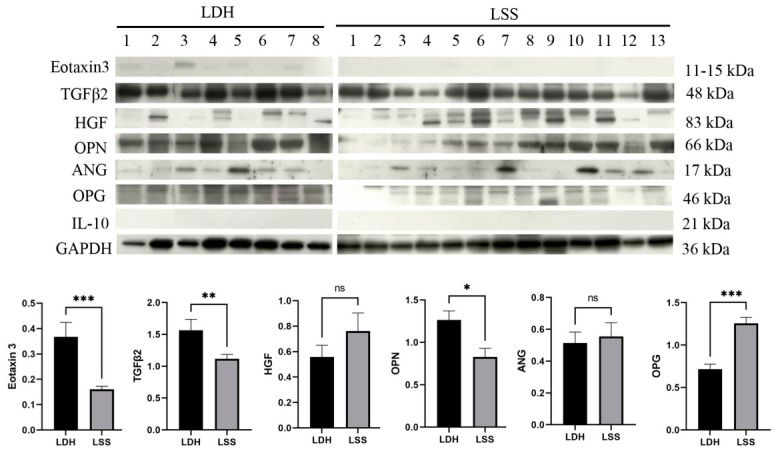
Western blot analysis of proteins that were significant from the array results. After obtaining the protein level, the blots were stripped and re-probed with GAPDH, and the expression levels were normalized against their own GAPDH. The levels were graphed and tested pair-wise. Those with a significant difference are labeled with a star (*: *p* < 0.05, **: *p* < 0.01. ***: *p* < 0.001. ns: not significant).

Protein array data suggested that eotaxin-3, HGF, FLT3-ligand, TGF-beta2, IL-7, and IL-10 levels in LDH were relatively higher than in LSS. The top expressors from the differentially expressed genes (DEGs) were angiogenin, osteopontin (gene name = SPP1), TIMP2, and osteoprotegerin (gene name = TNFRSF11B). The results from cytokine arrays were validated by Western blot using another batch of patient LFs, with 8 patients for LDH and 13 for LSS. The eotaxin-3, TGF-beta2, OPN, and OPG results corroborated the array data. IL-7 and IL-10 were not detected by Western blot. HGF and Ang levels did not differ significantly between the two groups when assessed using a Western blot (Figure 8).

### 3.7. LF Thickness

The pre-surgery LF thickness of patients whose tissues were used for the Western blot analysis was analyzed. The average LF thickness in the LDH group was 3.07, while in the LSS group it was 4.13. These were significantly different (*p* = 0.00221) (see Supplementary File S7 for details).

## 4. Discussion

**Figure 9:**
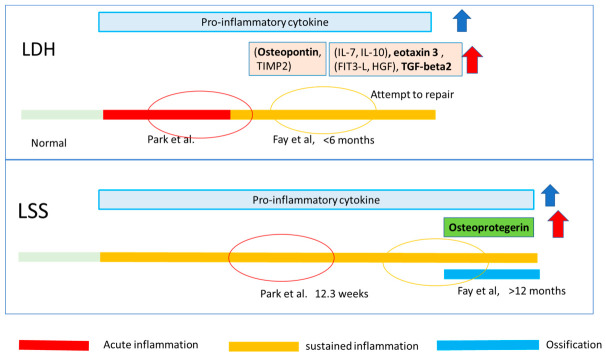
The proposed working model. The LF tissue from both LDH and LSS patients showed elevated levels of pro-inflammatory cytokines. The LF tissue from LDH patients expressed higher levels of angiogenic proteins and later markers known for immune regulation and tissue repair, whereas the LF tissue from LSS patients exhibited a fibrotic LF phenotype with lower protein levels for tissue repair and higher OPG expression. Park et al.’s results are depicted as representing an acute inflammatory stage in LDH and chronic inflammation in LSS for comparison. Upward arrow indicates up-regulation of protein levels.

This study compared the cytokine profiles and histology of LF tissue and other bio-samples from patients with LDH and LSS. The proteins IL-7, IL-10, eotaxin-3, Flt3L, HGF, and TGF-β2 showed elevated trends in LF tissue of LDH patients compared to that of LSS patients; a gene function analysis revealed that ANG, osteopontin, and TIMP2 were the top expressors in LDH, while in LSS, the top expressors were OPG, TIMP1, and CCL15. Elevated OPG levels were found in primary cultured LF cells from LSS patients, but were lower in those from LDH patients. Higher levels of TGF-β2 and eotaxin-3 in LDH tissue and higher OPG levels in cultured LSS cells were confirmed by independent Western blot analysis. The serum cytokine profile showed a trend toward elevated leptin in LSS patients compared with LDH patients. Many pro-inflammatory cytokines were elevated in both groups but there was no difference between the two groups. Taken together, the data from our study as well as others suggests a sustained inflammation paradigm and divergence by trend in tissue repair in LDH patients and a trend toward osteogenesis in LSS patients. The hypothesis proposed is illustrated in Figure 9 and explained as follows.

Some of the Western blots did not corroborate the protein array data. We believe that this partial concordance reflects several factors. First, the small sample size in the discovery phase likely led to unstable ranking of candidate proteins. Second, methodological differences between protein arrays and Western blotting—including antibody specificity, epitope recognition, detection range, and sensitivity to protein isoforms or processed forms—may contribute to discrepancies. Third, and importantly, the use of human ligament tissue introduces substantial biological heterogeneity across individuals, which may further reduce the reproducibility of marginal signals.

### 4.1. Tissue Array Analysis Identifies Proteins Potentially Involved in Repair in LDH, Requiring Further Validation

The current study is consistent with the previous literature, showing that hypertrophied LF expressed pro-inflammatory mediators, including COX-2, TNF-α, IL-1β, IL-8, and IL-15 [16]. COX-2 catalyzes the conversion of arachidonic acid into prostaglandin precursors and thereby contributes to inducible PGE_2_ production and inflammatory responses. Its enzymatic activity has been evaluated by measuring PGE_2_ production in tissue homogenates or explant-conditioned medium in the presence and absence of a selective COX-2 inhibitor [17,18]. However, the method we employed used antibodies to detect the presence of antigens, and the presence of this protein does not indicate activity of cox2 and downstream reaction. Because of this, our array, although detecting many inflammatory cytokines, does not detect the presence of the Cox2 or PGE2 proteins. The elevation of many pro-inflammatory cytokines provides grounds for further study of the cox2 enzyme activity using a more specific method.

Unlike some earlier studies that reported elevated IL-6 expression in degenerative spinal disease, we observed no intergroup difference in IL-6 in either serum or LF tissue [16,19]. Although not detected by Western blot due to their low expression level, we observed higher levels of IL-7 and IL-10 in LDH tissue when using the array method, which might suggest a stage-dependent immunoregulatory status. IL-7 promotes T-cell survival, homeostasis, and lymphopoiesis and has been suggested to function in the stimulation or recovery of T-cell populations, possibly after lymphodepletion or stress [20,21]. IL-10 is a classically anti-inflammatory cytokine. Its suppression of Th1 and macrophage activation indicates a shift toward immune regulation or resolution of inflammation [22]. Its gene expression was previously reported in the serum of patients, as well as in ossified or hypertrophic spinal ligaments, and was proposed to counterbalance local inflammation [23].

Eotaxin-3 (CCL26) is a well-established, potent chemoattractant that binds selectively to the CCR3 receptor, which is highly expressed on eosinophils (as well as basophils and Th2 cells). The expression of eotaxin-3 (CCL26) is strongly upregulated by classic Th2 cytokines like interleukin-4 (IL-4) and interleukin-13 (IL-13), establishing a direct link to a type 2 immune profile. While historically seen strictly as inflammatory/allergic effector cells, both Th2 environments and eosinophils are heavily implicated in driving downstream tissue modeling, cell proliferation, and wound healing or repair processes following tissue injury [24,25].

### 4.2. TGF-Beta Is Necessary but Not Sufficient for LSS Hypertrophy?

TGF-β2 can be immunosuppressive, promoting regulatory T-cells (Tregs) and tissue remodeling in certain environments, but it can also be pro-inflammatory. TGF-β signaling (often attributed to TGF-β1) is a canonical driver of fibrosis in LF [26]. Its expression could imply induction of tolerance, fibrosis, or wound-healing pathways [27]. Interestingly, we observed higher levels of TGF-β2 in the LDH group compared with the LSS group. One possible explanation relates to differences in disease duration at the time of surgery. In our cohort, the interval from symptom onset to surgery in LDH patients was generally less than 6 months, whereas LSS cases typically had a much longer clinical course, often exceeding 12 months and, in some cases, extending to 60–120 months (Figure 9). Thus, the LDH samples analyzed in this study may represent a subacute phase, in contrast to previously reported cohorts that may have included more acute presentations [14,28]. For example, Park et al. reported a mean duration from symptom onset to surgery of 12.3 weeks (range: 1–32 weeks) for LSS, although comparable information for LDH was not provided. It is therefore possible that differences in disease stage contribute to the observed discrepancies in TGF-β levels across studies.

The elevated TGF-β2 levels in LDH patients, together with the presence of several proteins associated with type II T-cell-related immune responses, may reflect a more active tissue remodeling or repair process in this group. In contrast, the LSS group, characterized by a more chronic disease course, may exhibit a reduced or altered repair response. These observations suggest that TGF-β signaling may play a context-dependent role, contributing to both physiological tissue maintenance and repair in earlier or less chronic stages, while also contributing to fibrotic processes in advanced disease. In other words, it is necessary but not sufficient for the development of LSS. Consequently, therapeutic strategies targeting TGF-β pathways should be approached with caution, as modulation of this pathway may have complex, potentially opposing effects depending on the disease stage.

### 4.3. OPG Expression: Is Its Upregulation Sufficient for LSS Etiology?

LSS tissue, as well as the primary cultured LF cells, from LSS patients expressed more OPG than that from LDH patients, and these results were verified independently with Western blot methods. OPG (TNFRSF11B) is a soluble decoy receptor for RANKL that blocks RANK signaling and osteoclastogenesis [29]. In bone-forming cells, canonical Wnt signaling increases OPG and decreases RANKL, shaping bone remodeling [30]. Genetic and clinical studies also link OPG to cervical spondylotic myeloradiculopathy and to ossification of the posterior longitudinal ligament (OPLL) [31,32]. The increased OPG may reflect a response to upstream signals (e.g., mechanical stress, TGF-β/IL-6) described in LF hypertrophy. The fact that LDH patients express lower OPG and have a thinner LF, while LSS patients express higher OPG and have a thicker LF, suggests a very high association of OPG with LSS. We observed that in most tissues, the ossification rate was very low (Figure 7D), indicating that the medical complaints arose well before overt bone formation in most LSS patients.

### 4.4. Dysregulation of Ligament Fibers Coincides with OPG Expression and Precedes Bone Formation

The deterioration of the LF ligament fiber follows a sequence: degeneration of fibers, infiltration of immune cells and neoangiogenesis, generation of chondroid tissue, calcification, and finally bone generation. When we looked for these pathologies, we found that degenerated fiber (for example, see Figure 7(B-b)) and neoangiogenesis and infiltration of immune cells (Figure 7(C-b,C-c)) were often associated with OPG expression (Figure 7(B-b1,C-b1,C-c1)), while OPG is downregulated in the calcification area (Figure 7(B-c1,C-d1)). In other words, alterations in ligament fiber organization are associated with OPG expression and occur prior to overt bone formation.

Osteoprotegerin (OPG; TNFRSF11B) is a secreted decoy receptor whose levels during osteogenesis are tightly controlled by osteogenic and inflammatory cues. Canonical osteogenic pathways, including TGF-β, BMP-2, and Wnt/β-catenin signaling, upregulate OPG expression in osteoblast-lineage cells, contributing to an anti-resorptive environment [33]. In contrast, pro-resorptive stimuli such as parathyroid hormone and inflammatory cytokines (e.g., TNF-α) suppress OPG while enhancing RANKL, thereby increasing the RANKL/OPG ratio that governs osteoclastogenesis. Beyond transcriptional control, OPG activity is modulated post-secretion through binding to heparan sulfate proteoglycans, thereby localizing the protein within the extracellular matrix and restricting its diffusion. Proteolytic processing by matrix metalloproteinases, including MMP-9, further regulates OPG bioavailability by reducing its capacity to neutralize RANKL. Thus, OPG function is determined not only by its expression level but also by its extracellular localization and stability.

Of these regulators, TGF-beta, BMP-2, Wnt/beta catenin, and TNF-alpha act on a very wide range of cell types and developmental stages and may not be good targets for treatment. Proteolytic processing by MMPs for OPG bioavailability might be an interesting target. The effect of the cleavage of OPG by MMPs or its upstream regulator TIMPs, which we discovered are expressed in high quantity and differentially expressed between LDH and LSS, for OPG bioavailability could be an interesting topic for further studies.

### 4.5. Serum Profile Suggests Increased Serum Pro-Inflammatory Cytokines May Be Necessary but Not Sufficient to Induce LSS

The serum samples showed that many pro-inflammatory cytokines in patients with LDH or LSS were not differentially expressed between the two groups. However, both were significantly elevated compared with the normal control (i.e., healthy volunteer) (Supplementary Files S5 and S6). Given this observation, it cannot be concluded that upregulated systemic inflammation is necessary for LSS, let alone sufficient to induce LSS. The expression levels of leptin, which consistently induced bone formation in cultured spinal ligament cells, differed but were not statistically significant (*p* = 0.41). This suggests that molecules regulating the metabolic pathways are crucial in LSS etiology, which corroborates previously reported findings.

### 4.6. Proposed Working Model

Integrating our findings with the prior literature, we propose a working model (Figure 9): (1) inflammation is long-lasting and persistent in both LDH and LSS; (2) growth/repair responses (most evident in LDH) include upregulation of regulatory cytokines eotaxin-3 (CCL26) and TGF-β2, within a pro-inflammatory background; (3) with chronicity (typical of LSS), the balance shifts toward matrix remodeling and fibrosis, accompanied by altered bone-related signaling and a higher OPG level; (4) depending on local signals, some patients may progress toward calcific/ossifying phenotypes, as demonstrated by further elevation of OPG and ossification [31]. Elevated OPG points to the RANKL/OPG axis as a potential biomarker of advanced remodeling.

## 5. Conclusions

LF tissue from both LDH and LSS patients showed elevated pro-inflammatory cytokines. The LF tissue of LDH patients showed higher levels of eotaxin-3 and TGF-β2, which are anti-inflammatory or pro-tissue-repair factors under these circumstances. The LF tissue from LSS patients exhibited a fibrotic LF phenotype with reduced protein availability for tissue repair and higher OPG expression, indicating commitment to bone formation. These data support a staged model of LF hypertrophy in which local cytokine dynamics coincide with progression from inflammation to reduced repair, fibrosis, and finally osteogenesis. The final osteogenesis is less prevalent in LDH patients.

## Figures and Tables

**Table 1:** Patient demographics.

	Total	Degeneration Type		*p* Value
		LDH	LSS	
Number	40	17	23	
Gender (male/female)	19/21	9/8	10/13	0.75
Age (years old)	54.8 ± 11.6	48.9 ± 11.9	59.1 ± 9.5	0.007 *
Diabetes	5	3 (17.6%)	4 (17.4%)	1.0
BMI	25.6 ± 3.7	24.6 ± 4.6	26.4 ± 2.7	0.15

BMI—Body Mass Index. *p* value—comparison between LDH and LSS. *—significant difference.

**Table 2:** Sample used for different preparation.

	Total	Degeneration Type	
		LDH	LSS
Tissue array	16	6	10
Serum array	12	5	7
Primary cell array	11	5	6
Western blot (primary cell)	11	4	7
Western blot (tissue)	21	8	13
Histological quantification	27	8	19

**Table 3:** Cytokines in LF (ligamentum flavum) tissue.

	LDH	LSS
IL-7	0.065 ± 0.08	0.008 ± 0.01
IL-10	0.19 ± 0.25	0.017 ± 0.02
Eotaxin-3	0.17 ± 0.11	0.06 ± 0.03
Flt-3-ligand	0.11 ± 0.08	0.05 ± 0.03
HGF	0.21 ± 0.11	0.1 ± 0.06
TGF-β2	0.4 ± 0.23	0.21 ± 0.1

IL—interleukin. HGF—hepatocyte growth factor. TGF—transforming growth factor.

## Data Availability

The original contributions presented in this study are included in the article.

## Supplementary Materials

The following supporting information can be downloaded at: https://www.mdpi.com/article/10.3390/cells15141278/s1, Supplementary file S1: multiple-comparison adjustment using FDR method-cultured cells; Supplementary file S2: multiple-comparison adjustment using FDR method-serum; Supplementary file S3: multiple-comparison adjustment using FDR method-tissue; Supplementary file S4: ranked gene expression list as the dataset in the STRING website; Supplementary file S5: Serum Cytokine Expression LDH vs. Normal, LSS vs normal; Supplementary file S6: Figure bar chart for serum cytokine expression LDH vs. LSS; Supplementary file S7: Average LF thickness LDH vs. LSS.

## Abbreviations

LDH	Lumbar disc herniation
LSS	Lumbar spinal stenosis
LF	Ligamentum flavum
OPG	Osteoprotegerin
RANKL	Receptor-activator-of-nuclear-factor-κB-ligand
IL	Interleukin
HGF	Hepatocyte growth factor
TGF	Transforming growth factor
H&E	Hematoxylin–eosin
CT	Computed tomography
MRI	Magnetic resonance imaging
HBSS	Hanks’ Balanced Salt Solution
FFPE	Formalin-fixed and paraffin-embedded
RPM	Revolutions per minute
DMEM	Dulbecco’s Modified Eagle Medium
MTT	3-(4,5-dimethylthiazol-2-yl)-2,5-diphenyl-2H-tetrazolium bromide
HEPES	4-(2-108 hydroxyethyl)-1-piperazineethanesulfonic acid
EDTA	Ethylenediaminetetraacetic acid
DTT	Dithiothreitol
PMSF	Phenylmethylsulfonyl Fluoride
HRP	Horseradish peroxidase
CCD	Charge-coupled device
DEG	Differentially expressed gene
PBS	Phosphate-buffered saline
DAB	3,3′-diaminobenzidine
SDS-PAGE	Sodium dodecyl sulfate–polyacrylamide gel electrophoresis
SEM	Standard error of the mean
AI	Artificial intelligence
TNF	Tumor necrosis factor
Flt3L	Flt-3-ligand
MF	Molecular function
BP	Biological process
OPN	Osteopontin
OPLL	Ossification of the posterior longitudinal ligament
