# Supplementary material for: Comparison of Cytokine Profiles in Ligamentum Flavum from Patients Undergoing Surgery for Lumbar Disc Herniation and Lumbar Spinal Stenosis: An Exploratory Study
Source: Cells. 2026 Jul 16;15(14):1278. doi: 10.3390/cells15141278 (PMC13406297; doi:10.3390/cells15141278)
Supplement: Supplementary file 1 [file cells-15-01278-s001.zip › supplementary file S7.pptx]

## Slide 1
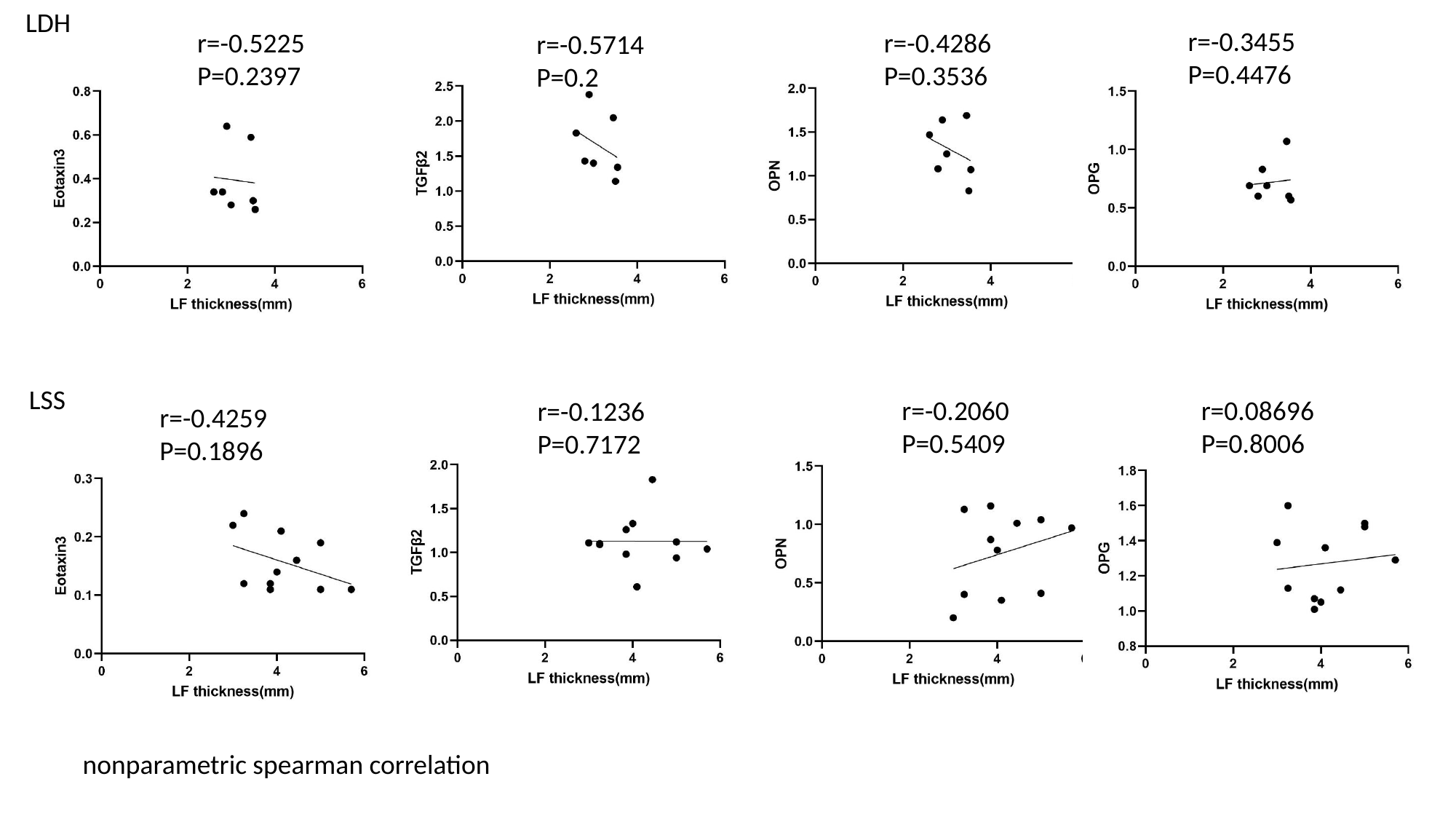

LDH
r=-0.3455
P=0.4476
r=-0.4286
P=0.3536
r=-0.5225
P=0.2397
r=-0.5714
P=0.2
LSS
r=-0.2060
P=0.5409
r=0.08696
P=0.8006
r=-0.1236
P=0.7172
r=-0.4259
P=0.1896
nonparametric spearman correlation

## Slide 2
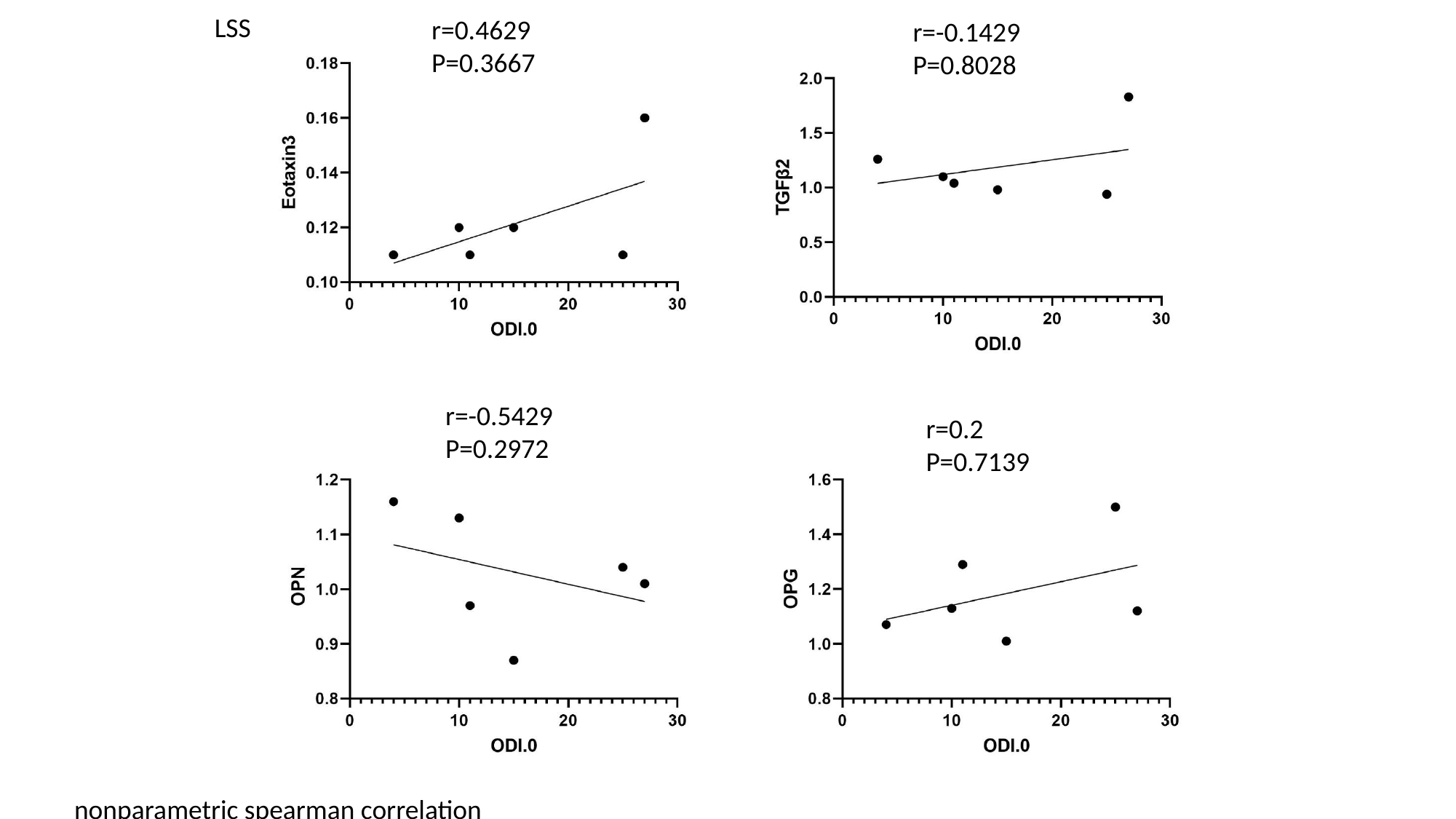

LSS
r=0.4629
P=0.3667
r=-0.1429
P=0.8028
r=-0.5429
P=0.2972
r=0.2
P=0.7139
nonparametric spearman correlation

## Slide 3
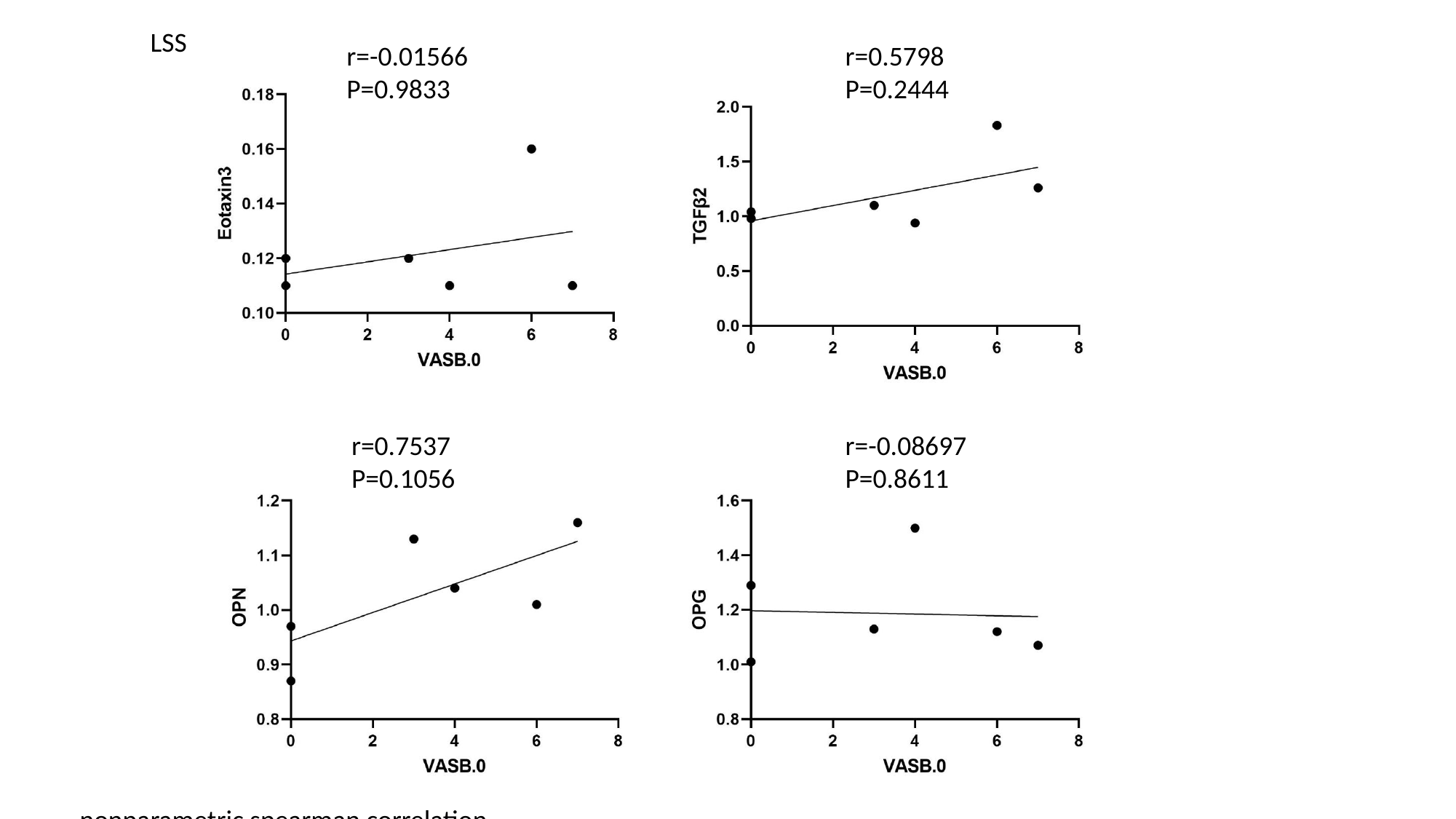

LSS
r=-0.01566
P=0.9833
r=0.5798
P=0.2444
r=0.7537
P=0.1056
r=-0.08697
P=0.8611
nonparametric spearman correlation

## Slide 4
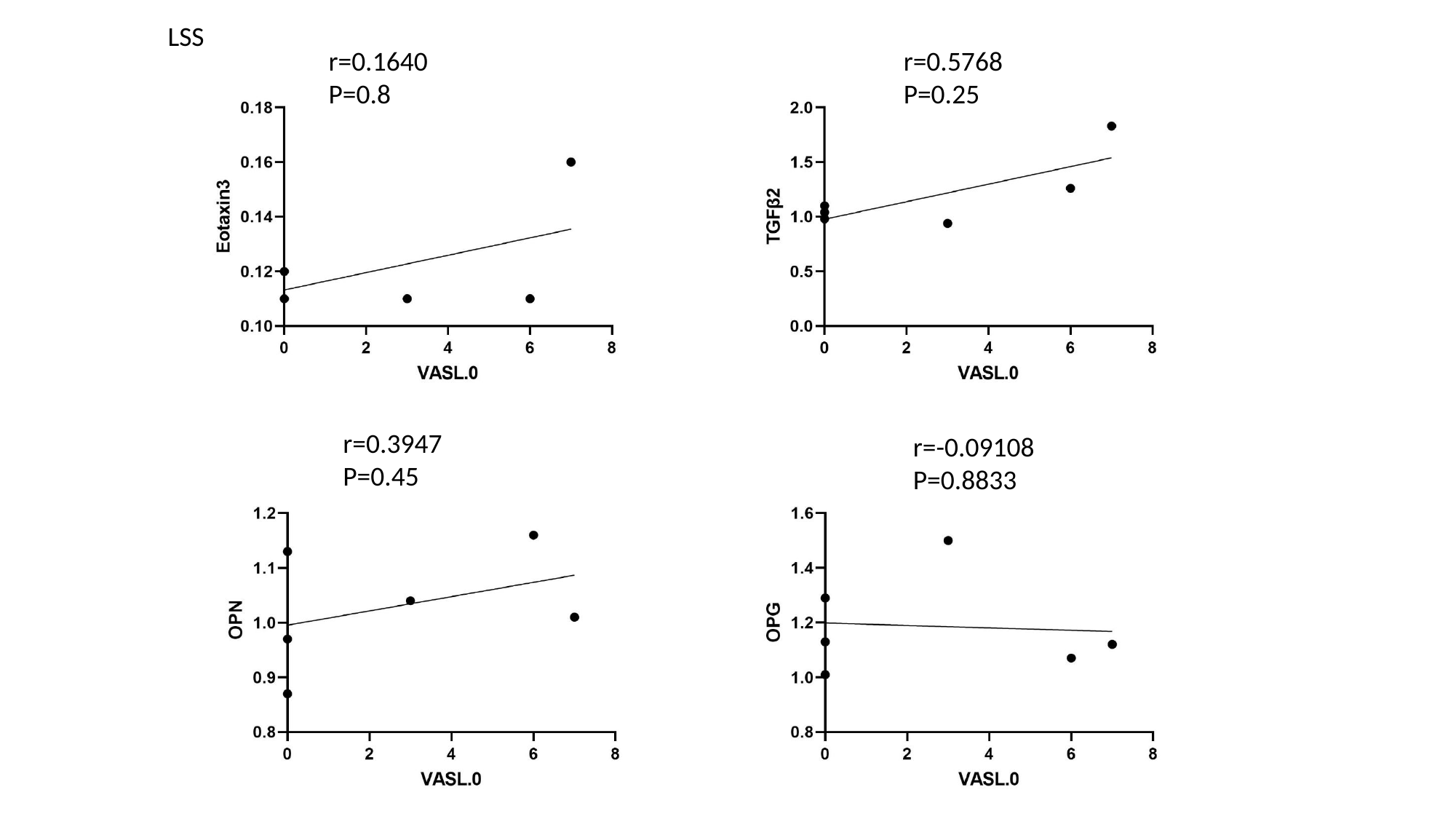

LSS
r=0.1640
P=0.8
r=0.5768
P=0.25
r=0.3947
P=0.45
r=-0.09108
P=0.8833
