# Supplementary figures and images for: Comparison of Cytokine Profiles in Ligamentum Flavum from Patients Undergoing Surgery for Lumbar Disc Herniation and Lumbar Spinal Stenosis: An Exploratory Study
Source: Cells. 2026 Jul 16;15(14):1278. doi: 10.3390/cells15141278 (PMC13406297; doi:10.3390/cells15141278)

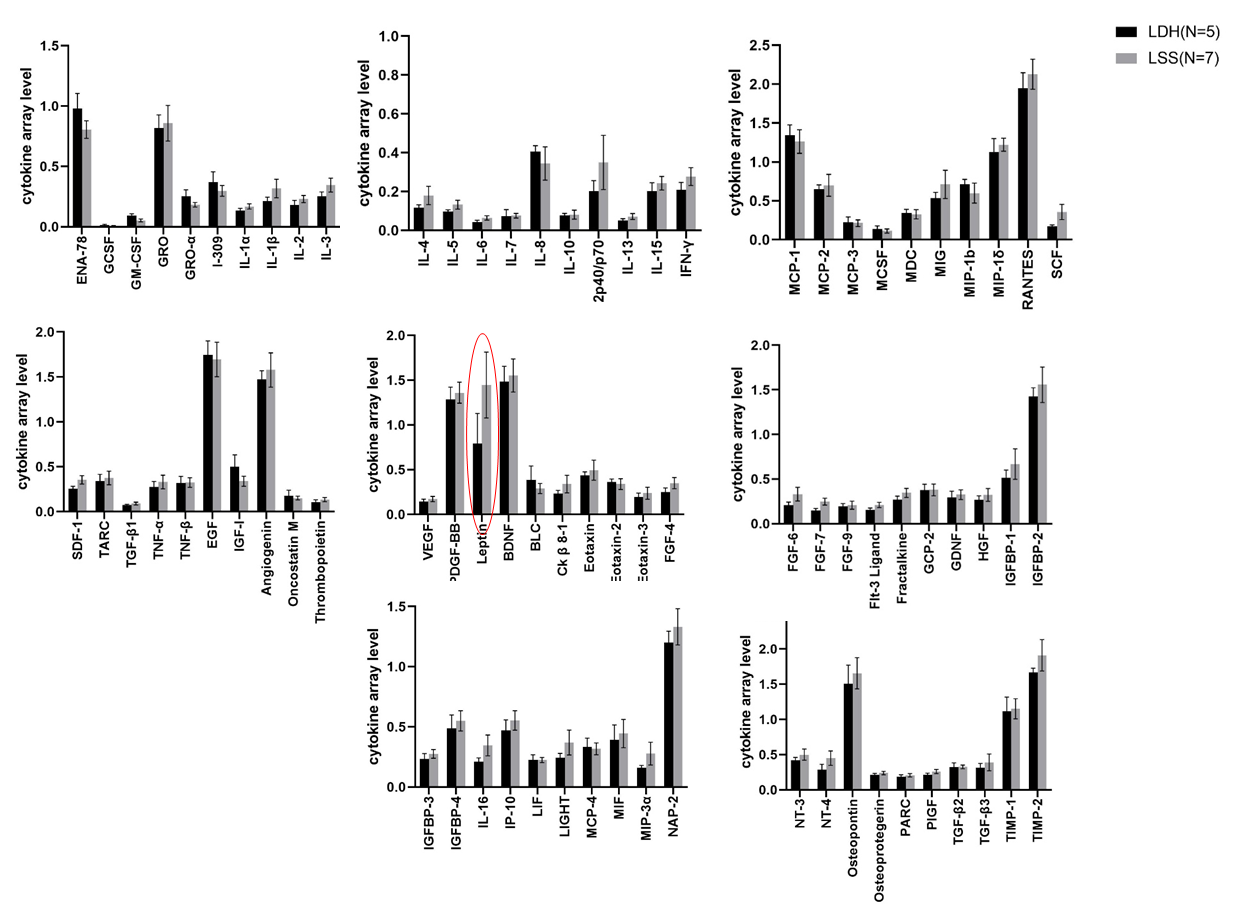

Supplement: Supplementary file 1 [file cells-15-01278-s001.zip › supplementary file S6.tif]
